# Comparative Chemistry of Propolis from Eight Brazilian Localities

**DOI:** 10.1155/2013/267878

**Published:** 2013-04-15

**Authors:** A. A. Righi, G. Negri, A. Salatino

**Affiliations:** ^1^Botany Department, Institute of Biosciences, University of São Paulo, São Paulo, SP, Brazil; ^2^Psychobiology Department, Federal University of the State of São Paulo, São Paulo, SP, Brazil

## Abstract

Propolis is a complex honeybee product with resinous aspect, containing plant exudates and beeswax. Their color, texture, and chemical composition vary, depending on the location of the hives and local flora. The most studied Brazilian propolis is the green (alecrim-do-campo) type, which contains mainly prenylated phenylpropanoids and caffeoylquinic acids. Other types of propolis are produced in Brazil, some with red color, others brown, grey, or black. The aim of the present work was to determine the chemical profiles of alcohol and chloroform extracts of eight samples of propolis, corresponding to six Brazilian regions. Methanol and chloroform extracts were obtained and analyzed by HPLC/DAD/ESI/MS and GC/MS. Two chemical profiles were recognized among the samples analyzed: (1) black Brazilian propolis, characterized chiefly by flavanones and glycosyl flavones, stemming from Picos (Piauí state) and Pirenópolis (Goiás state); (2) green Brazilian propolis, characterized by prenylated phenylpropanoids and caffeoylquinic acids, stemming from Cabo Verde (Bahia state), Lavras and Mira Bela (Minas Gerais state), Pariquera-Açu and Bauru (São Paulo state), and Ponta Grossa (Paraná state). The present work represents the first report of prenylated flavonoids in Brazilian propolis and schaftoside (apigenin-8-*C*-glucosyl-6-*C*-arabinose) in green propolis.

## 1. Introduction

Propolis is a resinous hive product containing bee secretions and plant resins. The wide diversity of plant species used by bees as resin sources for propolis production determines its chemical diversity. More than 300 constituents have already been identified [1], among which phenolic compounds such as flavonoids, phenolic acids, and phenolic acid esters have been reported as major constituents of propolis from the temperate zone [2].

The use of propolis with medicinal purpose dates back to 300 BC [3] and has been extensively used in folk medicine in the east and the west for centuries. It has been established that propolis possesses a wide spectrum of biological activities, such as antibacterial [4, 5], anti-inflammatory [6, 7], antioxidant [8, 9], hepatoprotective [10], and antitumoral [11]. The medicinal properties of propolis are due to a large variety of substances of plant origin, acting alone and/or synergistically. The high medicinal value of many propolis constituents stimulates chemical studies also of the corresponding plant sources [12].

The classes of propolis constituents include fatty and phenolic acids and esters, substituted phenolic esters [13], flavonoids (flavones, flavanones, flavonols, dihydroflavonols, chalcones) [14–16], mono-, sesqui-, di-, and triterpenes, steroids, aromatic aldehydes and alcohols, and naphthalene and stilbene derivatives [17, 18]. Propolis from temperate regions (poplar derived) contains mostly flavonoids, aromatic acids, and their esters [19]. Mediterranean propolis from Croatia, Algeria, Greece, and Cyprus has a poplar-type chemical profile, while samples from South Greece and Crete are rich in diterpenes [20]. Propolis from Taiwan and Okinawa contains prenylated flavanones as major constituents [21, 22], while propolis from Australia is rich in prenylated stilbenes [18]. Propolis from tropical regions contains a diversity of phenolics: prenylated cinnamic acid derivatives, flavonoids, polyprenylated benzophenones and lignans, and other classes of constituents [23].

Among tropical countries, Brazil has the widest chemical diversity of propolis types. Brazilian green propolis is the most abundantly produced and consumed, either internally or externally. It contains mostly phenylpropanoids, prenylated phenylpropanoids (e.g., artepillin C), and sesqui- and diterpenoids and is produced with material obtained from apices of *Baccharis dracunculifolia* (Asteraceae) [24]. Chalcones, pterocarpans, and other isoflavonoids are the main constituents of Brazilian red propolis, which depends on *Dalbergia ecastaphyllum* (Leguminosae, Faboideae) as the main resin source [25, 26]. Propolis produced in the Brazilian Amazon may contain predominantly polyprenylated benzophenones, probably derived from *Clusia *spp. [27]. While it seems certain that a specific species plays the role as the main resin provider, probably bees often use several plant sources for propolis production [28]. For example, Brazilian red propolis may contain isoflavonoids (from *Dalbergia*) and polyprenylated benzophenones (probably from *Clusia*) [25].

Notable differences are often found between propolis samples, not only from distant but also from nearby locations, and sometimes in the same locality. This holds either for European [29, 30] or green propolis [31–33], even restricting the analysis to samples of ‘‘typical” green propolis [34]. Among samples of green propolis there seems to be a gradual variation in the proportion of mevalonate-derived substances (terpenoids, including sesqui- and triterpenoids) and the typical shikimate-derived (phenolics, prenylated or not) compounds [34]. For example in the distributional area of Brazilian green propolis (Southeast Brazil) there are samples from different localities, some with deep green color, others dark, and others black; often, the green samples contain high level of phenolic compounds, while the dark and black ones contain mostly triterpenoids (unpublished observations).

Powerful chromatographic tools, such as HPLC-DAD-ESI-MS^*n*^ and GC/MS, are essential for the analysis of products comprising of complex mixtures, enabling the identification and quantification of their biologically active constituents [35]. The aim of this work was to characterize the chemical profile of samples from distant Brazilian localities, with the expectation to establish chemical affinities among the analyzed samples and detect constituents not commonly reported for Brazilian propolis.

## 2. Material and Methods

### 2.1. Propolis Sampling and Processing

**Figure 1:**
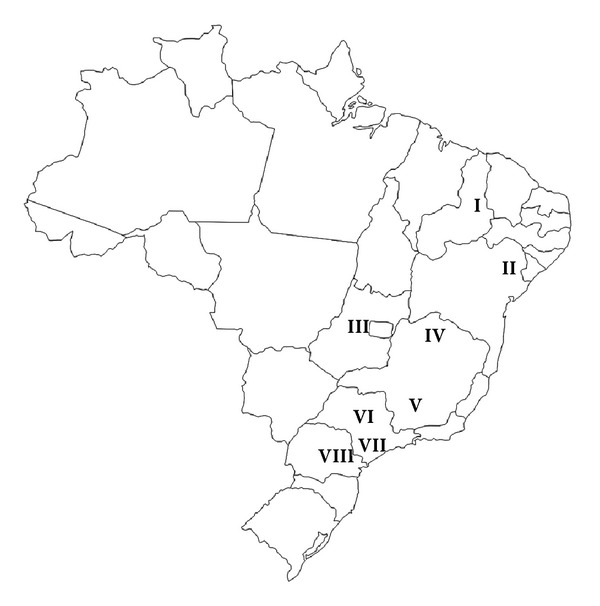
Brazilian map indicating the localities of the analyzed Brazilian propolis samples: **I**: Picos-PI; **II**: Cabo Verde-BA; **III**: Pirenópolis-GO; **IV**: Mira Bela-MG; **V**: Lavras-MG; **VI**: Bauru-SP; **VII**: Pariquera-Açu-SP; **VIII**: Ponta Grossa-PR.

Samples of propolis produced by *Apis mellifera* were obtained from eight Brazilian localities: Picos (state of Piauí, North Central Brazil) (black propolis), Cabo Verde (Bahia state, northeast) (green propolis), Pirenópolis (Goiás state, Central Brazil) (black propolis), Lavras and Mira Bela (Minas Gerais state, Southeast) (green propolis), Pariquera-Açu and Bauru (São Paulo state, southeast) (green propolis), and Ponta Grossa (Paraná state, south) (green propolis) (Figure 1). The samples were grounded with a mortar and pestle to a fine powder. Extracts were successively prepared in Soxhlet for 3 h with 5 g of each propolis sample, first with chloroform and then with methanol. Each extract was concentrated under reduced pressure and the residue evaporated on a steam bath to constant weight. The chloroform extracts were dissolved in diethyl ether and treated with diazomethane [36]. The dried methanol extracts were dissolved in methanol at 0.1 mg mL^−1^ prior to HPLC analysis.

### 2.2. Total Polyphenol Contents

Total polyphenol contents were determined according to the Folin-Ciocalteu colorimetric method [37], using *p*-coumaric acid as reference. The analyses were carried out in triplicates of each sample.

### 2.3. Total Flavonoid Contents

Total flavonoid contents were determined by the aluminium chloride [37] and dinitrophenylhydrazin [38] methods. Calibration curves were made using quercetin (aluminium chloride method) and pinocembrin (dinitrophenylhydrazin method) as reference. Total flavonoid contents were assumed to be the sum of the values obtained by each method. Analyses were performed in triplicates.

### 2.4. GC/EIMS Analysis

The diethyl ether solutions of the diazomethane-treated chloroform extracts were diluted to the 1000 ppm concentration. Ether solutions (1 *μ*L) of each extract was injected into a Hewlett Packard 5890 series II plus gas chromatography coupled to Chem Station System Mass Spectrometer 5989B operating with the EI mode at 70 eV. The GC conditions were as follows: DB-5HT fused silica capillary column (30 m × 0.32 mm internal diameter, 0.25 *μ*m film thickness) held at 100°C for 1 min and then heated to 300°C at 6°C min^−1^, the final temperature being held constant for 2 min; He was used as a carrier gas with flux of 1.5 mL min^−1^, linear velocity of 63 cm s^−1^, total flow 77,3 mL min^−1^, and split mode and solvent cut time of 3.0 min. The MS conditions were as follows: ionization voltage, 70 eV; filament current, 0.3 mA; detector voltage, −0.7 kV; MS scan range, 40–800 *m/z*. Injector and detector temperatures were at 300°C. The characterization of the constituents was based on comparison of corresponding mass spectra with data from the libraries Wiley-275 (Hewlett Packard), Wiley/NBS, and McLafferty and Stauffer [39].

### 2.5. HPLC/DAD/ESI/MS Analysis

Methanol extracts (10 *μ*L) were analyzed by HPLC/DAD/ESI/MS and HPLC/DAD/ESI/MS/MS. DAD SPD-M10Avp Shimadzu equipped with a photodiode array detector coupled to Esquire 3000 plus, Bruker Daltonics mass spectrometer via an electrospray ionization (ESI) source. The analyses were controlled by a computer running the Esquire NT Software from Bruker Daltonics. The diode-array detector was set at 270 nm, and the online UV spectra were recorded in the range of 250–360 nm. A reverse phase C18, Zorbax-5B-RP-18 (Hewlett Packard) column (4.6 × 250 mm, 5 *μ*m), and different linear gradients were used for analysis of each sample. The mobile phases consisted of eluent A (0.1% aq. HOAc) and eluent B (methanol). For the Ponta Grossa sample, the following gradient program was used: 0 min, 5% B; 10 min, 10% B; 16 min, 30% B; 72 min, 86% B. For the Picos, Pirenópolis, Lavras, Bauru, and Pariquera-Açu samples the following program was used: 0 min, 10% B; 12 min, 35% B; 27 min, 38% B; 50 min, 50% B; 100 min, 100% B. For the Cabo Verde sample, the program was 0 min, 10% B; 12 min, 20% B; 52 min, 40% B; 75 min, 86% B. For the Mira Bela sample, the program was 0 min, 5% B; 10 min, 10% B; 16 min, 34% B; 60 min, 78% B. The flow rate was 0.5 mL min^−1^ and the temperature of the column was maintained at 28°C. Negative-ion ESI was performed using an ion source voltage of −40 V and a capillary offset voltage of 4500 V. Nebulization was aided with a coaxial nitrogen sheath gas provided at a pressure of 27 psi. The dry gas temperature was set to 130°C and a dry gas flow of 4 L min^−1^ was used. Desolvation was assisted using a counter current nitrogen flow set at a flux of 7.0 L min^−1^ and a capillary temperature of 320°C. Mass spectra were recorded over the range 50–900 *m*/*z*. Mass spectrometry (MS/MS) data were acquired in the negative ionization mode. Compounds were identified by comparison of their UV and ESI/MS and ESI/MS/MS spectra with the literature data.

### 2.6. Clustering Analysis

Compounds identified by GC/MS and HPLC/MS of all eight propolis samples were analyzed using the neighbor-joining method and the software PAUP v.4.0b10 [40].

## 3. Results and Discussion

A wide variety of total phenols (0,9% to 27,3%) and total flavonoids (0,3% to 4,4%, Table 1) were observed. It is known that the amount of phenolic and flavonoid constituents varies widely according to propolis types and seasonal factors [24, 28]. The highest amounts of both total phenolic substances and flavonoids were obtained with propolis from Pirenópolis (27.341% and 4.432%, resp.) and Cabo Verde (25.867% and 3.148%, resp.) (Table 1). The main constituents found in the Pirenópolis sample were flavone-*C*-glycosides, prenyl flavonols, and rhamnetin, while in the propolis sample from Cabo Verde only flavone-*C*-glycosides and a minor content of caffeoylquinic derivatives were detected. Samples from Bauru, Ponta Grossa, and Lavras have intermediate contents of total phenolics (17.632%, 12.892%, and 9.281%, resp.) and showed the presence of caffeoylquinic derivatives, phenylpropanoids, and flavones. Finally, samples from Picos, Pariquera-Açu, and Mira Bela presented lower contents of total phenolics (5.620%, 2.66%, and 0.91%, resp.) and higher caffeic acid glucoside. In South America, the contents of phenols reported for Brazilian propolis have been higher than the values obtained from samples of other countries: Argentina (0,3%–5,5%), Uruguay (1,1%–3,7%), Chile (1,1%–4,3%), Peru (less than 0,1%), and Paraguay (0,3%) [14].

On the other hand, samples from Bauru, Picos, Pariquera-Açu, Ponta Grossa, Lavras, and Mira Bela showed low contents of total flavonoids: 1.97%, 1.243%, 0.884%, 0.859%, 0.685%, and 0.311%, respectively. It is interesting to note that all samples, except from Bauru, present flavone *C*-glycosides among their constituents. However, only the mentioned sample exhibited high content of caffeic acid ethyl phenyl ester derivatives. Among the samples studied in the present work, there seems to be a direct correlation between the contents of total phenols and total flavonoids (Table 1).

A high diversity of phenolic and nonphenolic substances was detected among the samples analyzed. Compounds **1**–**52** were present in less polar (chloroform) extracts and were detected and characterized by GC/EIMS analysis (Table 2). Several substances detected are wax constituents: long carbon chain fatty acids (**8**, **11**, **13**, **15**, **32**, and **34**) and *n*-alkanes (**33**, **36**, and **37**). Hydrocarbons and fatty acids have been pointed out as major constituents of wax propolis [36, 41]. Other detected substances are phenolic compounds, many of them prenylated. Such substances have been pointed out as constituents of *Baccharis dracunculifolia* and/or Brazilian green propolis: **1**, **3**, **5**, **9**, **10**, **14**, **24**, **25**, **28**, and **35** [12, 24, 42]. Compounds **3** and **14** (artepillin C) have been assigned the role as chemical markers of green propolis [12]. Terpenoids are frequent propolis constituents. Among them, diterpenoids (**17**, **19**, and **23**) have been reported for Brazilian propolis [43]. Triterpenoids (**40**, **43**–**52**) are also frequent constituents of Brazilian propolis (see, e.g., [24, 42]). Steroids, such as ergosterol (**42**), are terpenoids rarely found in propolis. A particularly interesting compound in Table 2 is the glyceride **39**, comprising of two residues of phenylpropanoid. Compounds such as this are constituents of *Populus* species and were obtained from a sample of Mexican propolis from the Sonora state [44], but the origin of **39** is certainly not a *Populus *plant.

Table 3 lists substances (**53**–**133**), mostly phenolic compounds, detected and characterized by HPLC/DAD/ESIMS. Caffeic acid (**73**) is very frequent in association with quinic acids or sugars. For example, in Table 3 several substances are caffeoylquinic acids, comprising of 1–3 residues of caffeic acid (**57**, **60**, **62**, **65**, **66**, **75**, **84**, **86**, **87**, **93**, **100**, **104**, **106**, and **107**). Such compounds are characteristic and abundant constituents of green propolis, obtained ideally from aqueous extracts [45, 46]. In some cases, one residue of caffeic acid is replaced by other phenylpropanoid, such as ferulic acid (**61**, **67**, **68**, and** 113**). In rare cases, quinic acid is esterified solely by ferulic acid (**72**). For the monocaffeoylquinic acids isomers, such as chlorogenic acids, the mass spectra in negative ion mode exhibited intense [M–H]^−^ ions at *m/z* 353.0 and some diagnostic fragments, due to caffeic (*m/z* 179) and quinic acid (*m/z* 191) moieties (**57**, **60**, **62**, **66**, and **75**). Di-*O*-(*E*)-caffeoylquinic acid positional isomers showed UV spectra identical to the monocaffeoylquinic acid derivatives and exhibited *m*/*z* 515.0 as deprotonated molecular ion, suggesting positional isomers of a quinic acid esterified with caffeoyl units at *m/z* 179, 191, and 353 [M–H–caffeoyl]^−^ (**84**, **86**, **87**, **93**, and **104**). Tricaffeoylquinic acid showed a deprotonated molecule at *m/z* 677.1 (**65**, **106**, and **107**). In general, tricaffeoylquinic acids with larger numbers of free equatorial hydroxyl groups in the quinic acid residue are more hydrophilic than those with larger numbers of free axial hydroxyl groups.

Glycosides are not frequent in propolis. Glycosides of caffeic acid (**54**, **58**, and **59**) have rarely (if at all) been reported as propolis constituents. *C*-glycosyl and *O*-glycosyl flavonoids were found in stingless bee honeys [47]. In the honey of *Apis mellifera* the contents of aglycone flavonoids typical of propolis (galangin, pinocembrin, quercetin) were found to be much higher than the contents of flavonoid glycosides. This has been attributed to a contamination of the honey by plant resins and propolis [48].

It has been recognized that flavonoids are not major constituents of Brazilian propolis, with the exception of isoflavonoids in red propolis [25]. Nonetheless, several flavonoid aglycones, as well as *O-* and *C*-glycosides, were detected in several among the eight samples of propolis in the present work: **56**, **64**, **69**–**71**, **74**, **76**–**83**, **88**–**92**, **97**–**99**, **101**–**103**, **105**, **108**, **109**, **111**, **114**–**116**, **118**, and **120**–**133**. The glycosylation of flavonoids generally occurs at the 7-hydroxyl in flavones and at the 3- and 7-hydroxyls in flavonols. The sugars usually correspond to hexoses (glucose, galactose, and rhamnose) and pentoses (arabinose and xylose). The carbon-carbon bond of *C*-glycosyl flavonoids is resistant to rupture and consequently fragments from the sugar moiety predominate in the mass spectra of *C*-glycosides.

Flavone derivatives presented UV spectra with *λ*_max⁡_ at 270.0 and 340.0 nm. The ESI-MS spectra of compound **79** (vicenin-2) showed deprotonated molecule [M–H]^−^ at *m*/*z* 593.0. The MS/MS spectrum in the negative ion mode produced ions at* m*/*z* 575.1 [M–H − 18]^−^, *m*/*z* 503.0 [M–H − 90]^−^ and a base peak at *m*/*z* 473.1 [M–H − 120]^−^, suggesting hexoses as sugar moieties. The fragment ions at *m*/*z* 353.4 [aglycone + 83]^−^ and *m*/*z* 383.2 [aglycone + 113]^−^ suggest that the aglycone is apigenin (270) + glucose + glucose, according to the literature data [49, 50]. The ESI-MS spectra of compound **81** (isoschaftoside-apigenin-8-*C*-glucosyl-6-*C*-arabinose), and compound **88** (schaftoside-apigenin-6-*C*-glucosyl-8-*C*-arabinose), showed identical deprotonated molecule at *m*/*z* 563.0 and exhibited similar fragmentation patterns: *m/z* 545.0 [(M–H) − 18]^−^, *m/z* 473.1 [(M–H) − 90]^−^, *m/z* 443.1 [(M–H) − 120]^−^, *m/z* 383.2 [aglycone + 113]^−^, and *m/z* 353.4 [aglycone + 83]^−^. In addition, they yielded the ion [M–H − 60]^−^ at *m/z* 503.0, generated by the fragmentation of a pentose in the MS/MS spectra, suggesting the presence of an arabinose moiety (−132 u), typical of asymmetric di-*C*-glycosylflavones. Isoschaftoside (**81**) showed a base peak at *m/z* 473 and a high abundance of the fragment at *m/z* 503.0, indicating the presence of a 6-*C*-pentosyl unit [51].

For *O*-glycosylated flavonoids, fragmentation pathway starts with the cleavage of the glycosidic bonds and elimination of the sugar moieties, with charge retention on the aglycone [52]. Tentative identification was based mainly on the MS data, UV-DAD spectra, and literature data.

Compounds **83** (luteolin-*O*-glucuronide - *m/z* 461.0) and **108** (apigenin-*O*-glucuronide - *m/z *445.1) both underwent loss of a glucuronide moiety with 176 u. Compound **90** (pentosyl-orientin) yielded a deprotonated molecule ion at *m/z* 579.0 and its MS/MS spectrum produced ions at *m/z* 327.0 [M–H − (120 + 132)]^−^ and the loss of 132 u corresponding to a pentose moiety. This fragmentation pattern is typical of *O*-glycosylated *C*-glycosyl flavones [53], indicating a pentose *O-*linked to a *C*-linked glucose.

Compound **99** (dimethoxy-naringenin-diglucoside) exhibited UV maximum absorption at 280.0 nm and a deprotonated molecule ion at *m/z* 623.4. Its MS/MS analysis produced ions at *m/z* 533.1 (M–H − 90.0)^−^, *m*/*z* 503.1 (M–H − 120.0)^−^, *m*/*z* 413.1 [aglycone + 113.0], and *m*/*z* 383.1 [aglycone + 83.0]. To our knowledge, this is the first report of this compound for propolis, having previously been identified from *Citrus *genotypes [54].

Among the constituents identified stand out the prenylated flavonols **109**, **125**, **126**, and **128**–**133**. Prenylated phenylpropanoids typical of Brazilian green propolis were also detected, such as compounds **95**, **96**, **117**, and **119** (artepillin C).

**Figure 2:**
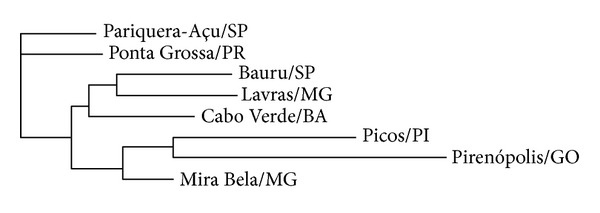
Clustering analysis of identified constituents by GC/MS and HPLC/ESI/MS of samples of Brazilian propolis using the neighbor-joining method.

The chemical affinities among the analyzed propolis samples are shown in Figure 2. One of the clusters of the dendrogram combines the samples from Bauru (SP, southeast), Lavras (MG, southeast), and Cabo Verde (BA, northeast). It is somewhat surprising the emergence in this cluster of the sample from the latter locality, which is much up north from the commonly admitted distribution of green propolis. The three samples share several prenylated phenylpropanoids (e.g., **9**, **14**, **24**, **25**, and **35**) typical of green propolis (Table 4). The sample from Cabo Verde stands apart in the clade for the more frequent possession of flavonoid glycosides, for example, **88**–**91**, **98**, **99**, and **101** (Table 4). Flavonoids are major compounds in propolis from temperate regions, derived from exudates from the vegetative buds of *Populus* spp. [19, 55]. However, flavonoids commonly detected in propolis are aglycones, and only rare flavonoid glycosides have been reported as propolis constituents.

The samples from Pariquera-Açu (SP, southeast) and Ponta Grossa (PR, south) contain also some of these green propolis constituents but emerge at the base of the clustering analysis tree (Figure 2). Both contain substances rarely or never reported for green propolis, such as flavonoid glycosides (e.g., **81**, **88**, and **97**; Table 4). They also contain or share diterpenes, such as **17** and **23** (Table 4), a class of substances known to occur in Brazilian propolis [43, 56, 57] and abundant in Mediterranean propolis [20, 58].

The samples from Mira Bela (MG, southeast), Pirenópolis (GO, Central Brazil), and Picos (PI, central-north) constitute another cluster, in spite of the large distances among the localities. The chemical composition of Mira Bela stands out by the total absence of nonpolar constituents from the chloroform extract (Table 4). It is worth noting that triterpenes (**42**–**52**; Table 4) were detected only in black propolis samples (from Picos and Pirenópolis). Besides this, both samples contain, and sometimes share, wax constituents, such as fatty acids (e.g., **32**, **34**) and *n*-alkanes (e.g., **33**, **36**, and **37**; Table 4). Flavonoids were more often detected in the samples from this cluster. Examples are the aglycones quercetin (**111**), isorhamnetin (**114**), and rhamnetin (**115**; Table 4). In addition, the sample from Mira Bela is characterized for containing flavone *C-*glycosides, such as **77**, **79**, **80**, and **82** (Table 4). The long branches corresponding to the samples of Picos and chiefly of Pirenópolis indicate a high number of exclusive characteristics. The sample from Picos is particularly characterized by flavonol-*O*-glycosides: **92**, **102**, **103**, and **105**. A remarkable characteristic of the sample from Pirenópolis is represented by prenylated flavones (**109**) and flavonols (**125**, **126**, and **128**–**133**; Table 4). The present work is the first report of prenylated flavonoids for Brazilian propolis. Prenyl-flavonoids are a rare feature in Neo-Tropical propolis but are characteristic in propolis derived from *Macaranga* spp., having been reported from Okinawa [59] and other localities, such as Egypt and Kenya [12]. Prenylation of aromatic compounds is a chemical feature enhancing biological activities [60]. Another interesting compound from the Pirenópolis sample is the glyceride **39** (Table 4). The frequent detection of flavonoids in the samples from Picos and Pirenópolis is coherent with the high amount of total flavonoids shown in Table 1.

## 4. Conclusions

Compounds derived from *Baccharis dracunculifolia* (alecrim-do-campo plant) are often found in Brazilian propolis, despite considerable chemical differences among them. Propolis samples from the typical distributional zone of alecrim-do-campo plant (southeast of Brazil) may have little or no chemical affinity at all with green propolis. On the other hand, samples from the northeast of Brazil, such as from Cabo Verde, may have high chemical affinity with green propolis. Analysis of propolis from distinct regions of Brazil is likely to provide chemical substances rarely found in propolis (such as flavonoid glycosides) and substances with high biological activity (such as prenylated flavonoids).

## Figures and Tables

**Table 1:** Contents of total phenols and flavonoids of samples of Brazilian propolis from localities in the states of Paraná (PR, south), São Paulo (SP, southeast), Minas Gerais (MG, southeast), Goiás (GO, Central Brazil), Bahia (BA, northeast), and Piauí (PI, central north).

Samples	Total phenols (%)	Flavonoids contents (%)		Total flavonoids (%)
		DNP method	AlCl_3_ method	
Pirenópolis (GO)	27.341 ± 0.074	0.800 ± 0.000	5.158 ± 0.002	4.432 ± 0.002
Cabo Verde (BA)	25.867 ± 0.045	0.368 ± 0.001	3.705 ± 0.004	3.148 ± 0.003
Bauru (SP)	17.632 ± 0.043	0.360 ± 0.000	2.131 ± 0.000	1.970 ± 0.000
Ponta Grossa (PR)	12.892 ± 0.022	0.367 ± 0.000	0.958 ± 0.002	0.859 ± 0.001
Lavras (MG)	9.281 ± 0.029	0.407 ± 0.000	0.740 ± 0.000	0.685 ± 0.000
Picos (PI)	5.620 ± 0.017	0.323 ± 0.000	1.427 ± 0.000	1.243 ± 0.001
Pariquera-Açu (SP)	2.660 ± 0.018	0.280 ± 0.000	1.005 ± 0.004	0.884 ± 0.004
Mira Bela (MG)	0.910 ± 0.005	0.206 ± 0.000	0.333 ± 0.000	0.311 ± 0.000

**Table 2:** Constituents from the chloroform extract of samples of Brazilian propolis, characterized by GC/EIMS.

Compounds		Rt (min)	Molecular ion
**1**	4-Vinyl phenol	4.2	120 (100, C_8_H_8_O^+^)
**2**	Dihydrocinnamic acid methyl ester	6.4	164 (40, C_10_H_12_O_2_^+^), 104 (100, C_8_H_8_^+^), 91 (60)
**3**	*p*-Vinyl-*O*-prenyl phenol	9.7	188 (50, C_13_H_16_O^+^), 133 (100, C_9_H_9_O^+^)
**4**	2-T-butylnaphto-[2,3-b]-furan,4,9-dione	13.5	254 (30, C_16_H_14_O_3_^+^), 239.0 (100, M–CH_3_)
**5**	*p*-Hydroxycinnamic acid methyl ester	13.5	178 (54, C_10_H_10_O_3_^+^), 147 (100, C_9_H_7_O_2_^+^), 119 (41, C_8_H_7_O^+^), 91 (60)
**6**	Methyl-*O*-benzoylbenzoate	13.7	240 (15, C_15_H_12_O_3_), 209 (17), 163 (100), 105 (78), 77 (73)
**7**	Benzene ethoxy	13.8	122 (13, C_8_H_10_O), 105 (16), 93 (70), 91 (24), 79 (32), 77 (21)
**8**	*n*-Hexadecanoic acid methyl ester	14.5	270 (3) (C_17_H_34_O_2_), 143 (12), 87 (53), 74 (100)
**9**	Allyl-3-prenylcinnamate	14.5	256 (64, C_17_H_20_O_2_^+^), 201 (84, C_13_H_13_O_2_^+^), 185 (100, C_12_H_9_O_2_^+^), 157 (60, C_12_H_13_^+^), 145 (95, C_11_H_13_^+^)
**10**	2,2-Dimethylchromene-6-propenoic acid methyl ester	15.5	244 (11), 229 (100), 144 (6)
**11**	Palmitic acid	16.4	256 (5, C_16_H_32_O_2_), 241 (8), 187 (14), 121 (51)
**12**	Pinobanksin methyl ether	16.6	286 (C_16_H_14_O_5_) (10), 271 (100)
**13**	Oleic acid methyl ester	16.7	296 (1) (C_19_H_36_O_2_), 264 (6)
**14**	4-Hydroxy-3-prenylcinnamic acid methyl ester	16.7	246 (70, C_15_H_18_O_3_^+^), 191 (100, C_11_H_11_O_3_^+^), 171 (20), 131 (23)
**15**	*n*-Stearic acid methyl ester	17.2	298 (1) (C_19_H_38_O_2_), 242 (7), 143 (18), 87 (70), 74 (100)
**16**	Ferulic acid octadiene ester	17.8	302 (20, C_18_H_22_O_4_), 287 (40), 271 (26), 257 (13), 246 (47), 231 (76), 203 (100), 187 (83)
**17**	Methyl dehydroabietate	17.8	314 (8, C_21_H_30_O_2_), 299 (10), 239 (100), 141 (14), 128 (14)
**18**	*n*-propyl-*P*-hydroxybenzoate	18.2	180 (5, C_10_H_12_O_3_), 161 (11), 121 (100), 105 (18), 91 (15), 77 (11)
**19**	12,16-Epoxy-6-hydroxy-(15-16)-abeo-5,8,11,13-abietatetraene-7-one	18.2	312 (17, C_20_H_24_O_3_), 241 (5), 237 (100), 197 (25)
**20**	Pentyl benzene	18.6	148 (16, C_11_H_16_), 133 (23), 121 (62), 105 (51), 91 (69), 77 (50)
**21**	Ferulic acid octene ester	19	304 (29, C_18_H_24_O_4_), 271 (22), 247 (8), 246 (100), 231 (68), 199 (23), 191 (21), 187 (42), 185 (12), 131 (36), 177 (28), 131 (36), 115 (30), 105 (14), 91 (23), 77 (19)
**22**	*n*-Phenyl hexadecane	19.2	302 (9, C_22_H_38_), 287 (10), 235 (8), 175 (31), 119 (51), 105 (61), 91 (77), 77 (63)
**23**	Ethyl dehydroabietate	19.5	328 (25, C_22_H_32_O_2_), 268 (9), 254 (17), 253 (100), 187 (24), 156 (12), 141 (14)
**24**	Hydroxy-diprenylcinnamic acid methyl ester (artepillin C)	20.6	314 (68, C_20_H_26_O_3_^+^), 259 (100, C_16_H_19_O_3_^+^), 243 (54, C_15_H_15_O_3_^+^), 211 (38), 203 (90, C_12_H_11_O_3_^+^)
**25**	2,2-Dimethyl-8-prenylchromene-6-propenoic acid methyl ester	20.7	312 (14), 297 (100)
**26**	Pinobanksin-5-methyl ether acetate	20.9	328 (28, C_20_H_24_O_4_^+^), 257 (89, M − 71), 313 (15)
**27**	Benzoic acid, 4(4-hydroxybenzoyl)-methylester	21.2	256 (6, C_15_H_12_O_4_^+^), 241 (9), 161 (12), 121 (100), 147 (11)
**28**	3-Hydroxy-2,2-dimethyl-8-prenylchromane-6-propenoic acid methyl ester	21.4	330 (100, C_20_H_26_O_4_^+^), 297 (30, C_19_H_21_O_3_^+^), 272 (50, C_17_H_20_O_3_^+^), 225 (60, C_15_H_13_O_2_^+^), 197 (50, C_14_H_13_O^+^), 171 (50)
**29**	Benzoic acid, 2-propoxy-(4-hydroxybenzoyl)-methyl ester	21.8	314 (3, C_18_H_18_ O_5_), 121 (100), 109 (21), 105 (25), 91 (30), 81 (47)
**30**	Benzylic alcohol, 2-propoxy-(4-hydroxybenzoyl)-methyl ester	22	316 (4, C_18_H_20_ O_5_), 301 (4), 257 (10), 189 (22), 147 (10), 121 (100), 105 (26)
**31**	Benzoic acid, 2(4-hydroxybenzoyl)-methyl ester	22.7	256 (10), 255 (50), 121 (100), 105 (33), 91 (44), 77 (35)
**32**	*n*-Docosanoic acid methyl ester	23	354 (4) (C_23_H_46_ O_2_), 87 (70), 74 (100)
**33**	*n*-Heptacosane	23.11	C_27_H_56_: *m/z* 380, C27
**34**	*n*-Tetracosanoic acid methyl ester	23.4	382 (4, C_25_H_50_ O_2_), 143 (14), 87 (60), 74 (100)
**35**	4-Dihydrocinnamoiloxy-3-prenylcinnamic acid methyl ester	24.7	378 (2, C_24_H_26_O_4_^+^), 246 (84, C_15_H_18_O_3_^+^), 105 (82, C_8_H_9_^+^), 91 (100)
**36**	*n*-Nonacosane	24.9	C_29_H_60_: *m/z* 408, C29
**37**	*n*-Hentriacontane	26.7	(C_31_H_64_): *m/z* 436, C31
**38**	Carbomethoxy benzyl caffeate ester	26.9	328 (60) (C_18_H_16_O_6_), 327 (50), 285 (40), 268 (33), 207 (40), 181 (40), 150 (40), 135 (100), 115 (40), 107 (30), 105 (30), 94 (30), 91 (45), 77 (60)
**39**	Dihydrocaffeoyl-dihydrocinnamoyl-glyceride	27.21	430 (9, C_23_H_26_O_8_), 399 (9), 380 (25), 281 (25), 232 (30), 180 (25), 175 (29), 161 (100), 105 (12), 95 (11), 93 (9), 81 (16), 77 (16)
**40**	**β**-Amyrin	28	426 (16, C_30_H_50_O^+^), 408 (C_30_H_48_^+^), 218 (100, C_16_H_26_^+^), 203 (40, C_15_H_23_^+^), 189 (25, C_14_H_21_^+^)
**41**	Propyl carbomethoxy benzyl dihydrocaffeate ester	28.1	372 (24, C_21_H_24_ O_6_), 371 (26), 357 (70), 314 (30), 299 (30), 207 (42), 167 (30), 135 (100), 107 (50), 91 (50), 93 (50), 95 (57), 77 (69)
**42**	Ergosterol	28.96	396 (16, C_28_H_44_O), 381 (26), 281 (35), 232 (35), 217 (34), 207 (19), 165 (10), 164 (27), 161 (30), 151 (39)
**43**	Lupenone	29.5	424 (7, C_30_H_48_O), 218 (50), 205 (100), 189 (50), 177 (50), 109 (56), 105 (24)
**44**	*β*-Amyrinone	30.5	424 (6, C_30_H_48_O), 218 (100), 205 (60), 203 (40), 189 (23)
**45**	*α*-Amyrinone	30.9	424 (6), 218 (100), 205 (60), 203 (20), 189 (23)
**46**	*α*-Amyrin	31	426 (6, C_30_H_50_O), 411 (15), 218 (100), 207 (90), 203 (18), 189 (24)
**47**	**β**-Amyrin acetate	31.6	468 (1, C_32_H_52_ O_2_), 218 (100), 203 (40), 189 (45)
**48**	**α**-Amyrin acetate	32.5	468 (1, C_32_H_52_ O_2_), 218 (100), 203 (20), 189 (35)
**49**	Taraxerone	33.4	424 (4), 205 (80), 189 (76), 109 (74), 69 (100)
**50**	Oleanene	33.9	410 (95), 395 (100, M–CH_3_), 174 (40), 165 (58)
**51**	Pteron-14-en-7-one	35.6	424 (66), 409 (100), 383 (64), 371 (56), 165 (50)
**52**	Olean-14-en-3,28-dione	36.2	440 (30, C_30_H_46_ O_2_), 425 (100), 409 (78), 397 (76), 385 (60), 183 (75), 165 (70)

**Table 3:** Constituents from the methanol extract of Brazilian propolis from Paraná (south), São Paulo and Minas Gerais (southeast), Goiás (central), Bahia (northeast), and Piauí (central-north) states, characterized by HPLC/DAD/ESI/MS.

Compound	Rt (min)	UV/DAD	[M–H]^−^	Proposed structure
**53**	4.9	270	181	Homovanillic acid
**54**	5.5	300, 330	341.2	Caffeic acid 4-*O*-glucoside
**55**	5.7	ND	191.1	Quinic acid
**56**	15	290	303.1	Dihydroquercetin
**57**	16.1	330, 300	353	Caffeoylquinic acid
**58**	16.3	300, 330	311	Caffeic acid 4-*O*-arabinoside
**59**	19.5	300, 330	311.2	Caffeic acid 4-*O*-xyloside
**60**	19.6	300, 330	353.1	Caffeoylquinic acid
**61**	20.3	330, 300	529	Feruloyl-caffeoylquinic acid
**62**	20.4	300, 330	353.3	Caffeoylquinic acid
**63**	21.9	ND	183	Methoxy-dihydroxy benzoic acid
**64**	22.1	270, 340	475.5	Dimethoxy-luteolin-glucoside
**65**	22.3	300, 330	677.1	Tricaffeoylquinic acid
**66**	22.3	330, 300	353.5	Caffeoylquinic acid
**67**	22.3	330, 300	529	Feruloyl-caffeoylquinic acid
**68**	22.7	330, 300	529	Feruloyl-caffeoylquinic acid
**69**	23.8	ND	607.3	Methylkaempferol-*O*-rutinoside
**70**	23.9	280, 330sh	433.3	Naringenin-*C*-glucoside
**71**	24	270, 338	577.5	Apigenin-*O*-rutinoside
**72**	24.3	300, 330	367.5	Feruloylquinic acid
**73**	24.9	ND	179.3	Caffeic acid
**74**	25.2	ND	435.1	Delphinidin arabinoside
**75**	25.7	330, 300	353.3	Caffeoylquinic acid
**76**	26.4	ND	421.2	Catechin arabinoside
**77**	27.1	270, 338	577.1	Apigenin-di-*C*-glucosyl rhamnoside
**78**	27.4	270, 338	415.3	Apigenin-*C*-rhamnoside
**79**	27.7	270, 338	593.1	Apigenin-6,8-di-*C*-glucoside (vicenin-2)
**80**	28.3	270, 338	547	Apigenin-*C*-rhamnosyl arabinoside
**81**	28.8	270, 340	563.5	Apigenin-6-*C*-glucosyl-8-*C*-arabinose (isoschaftoside)
**82**	29.3	270, 340	415.4	Apigenin-*C*-rhamnoside
**83**	29.6	270, 338	461	Luteolin-*O*-glucuronide
**84**	33.6	300, 330	515.4	Dicaffeoylquinic acid
**85**	35.8	330, 300	315	Caffeic acid-dihydroxy phenyl ethyl ester
**86**	36	300, 330	515.8	Dicaffeoylquinic acid
**87**	37.2	330, 300	515	Dicaffeoylquinic acid
**88**	38.6	270, 340	563.6	Apigenin-8-*C*-glucosyl-6-*C*-arabinose (schaftoside)
**89**	41.4	270, 340	609.4	Luteolin-6,8-di-*C*-glucoside (lucenin-2)
**90**	42.7	270, 340	579.3	Pentosyl orientin
**91**	42.8	270, 340	431	Vitexin
**92**	44.3	ND	463.3	Quercetin-*O*-glucoside
**93**	45.8	ND	515.3	Dicaffeoylquinic acid
**94**	48.4	330, 300	375.1	Ferulic acid-methoxy trihydroxy phenyl ethyl ester
**95**	49.1	310	231.2	Hydroxy-prenylcinnamic acid
**96**	49.8	310	315.4	3-Prenyl-4-(2-methylpropionyloxy)-cinnamic acid methyl ester
**97**	52.3	270, 340	489.8	Luteolin acetylglucoside
**98**	52.5	270, 340	461.2	Chrysoeriol-*C*-glucoside
**99**	53.1	280	623.4	Dimethoxy naringenin-diglucoside
**100**	53.8	300, 330	529.7	Caffeoylferuloylquinic acid
**101**	54.1	270, 340	593.3	Apigenin-di-*O*-glucoside
**102**	54.6	256, 356	433	Quercetin-*O*-arabinoside
**103**	55.5	260, 355	447.1	Quercetin-*O*-rhamnoside
**104**	56.3	300, 330	515.9	Dicaffeoylquinic acid
**105**	56.6	260, 350	477.1	Isorhamnetin-glucoside
**106**	58.8	300, 330	677.4	Tricaffeoylquinic acid
**107**	59.8	330, 300	677.3	Tricaffeoylquinic acid
**108**	60.4	270, 335	445.1	Apigenin-*O*-glucuronide
**109**	60.5	ND	389.3	Diprenyl chrysin
**110**	60.6	330	329	Ferulic acid dihydroxy phenyl ethyl ester
**111**	65.9	260, 353	301.2	Quercetin
**112**	66	ND	309.1	Cinnamoyl hexoside
**113**	66.5	330, 300	529.6	Caffeoylferuloylquinic acid
**114**	67.9	256, 356	315.1	Isorhamnetin
**115**	70.6	256, 356	315.1	Rhamnetin
**116**	71.2	255, 354	331	Laricitin
**117**	72.1	310	315.5	3-Hydroxy-2,2-dimethyl-8-prenylchromane-6-propenoic acid
**118**	72.3	230, 350	299.1	Methyl licochalcone B
**119**	73	310	299	Hydroxy-diprenylcinnamic acid (artepillin C)
**120**	73.9	256, 356	329.1	Quercetin-dimethyl ether
**121**	74.2	ND	387.4	Pentamethoxy hydroxy flavonol
**122**	76.9	ND	371.6	Pentamethoxy flavonol
**123**	78	ND	401.2	Nobiletin
**124**	79.1	ND	399.5	Chrysin rhamnoside
**125**	82.1	ND	395.2	Prenyl-trimethoxyluteolin
**126**	83.2	255, 355	383.4	Prenyl-methoxyquercetin
**127**	86	260, 370	273.1	Phloretin
**128**	87.5	260, 360	383.3	Prenyl-methoxyquercetin
**129**	88.1	260, 360	367.1	Prenyl-methoxykaempferol
**130**	88.9	ND	397.2	Prenyl-dimethoxyquercetin
**131**	89.9	260, 355	381.2	Prenyl-dimethoxykaempferol
**132**	91.1	260, 350	381.8	Prenyl-dimethoxykaempferol
**133**	93.1	260, 360	395.2	Prenyl-trimethoxykaempferol

**Table 4:** Constituents from the chloroform and methanol extracts of samples of Brazilian propolis from the municipalities of Pariquera-Açu (Par; state of São Paulo), Ponta Grossa (PGr; state of Paraná), Bauru (Bau; state of São Paulo), Lavras (Lav; state of Minas Gerais), Cabo Verde (CVe; state of Bahia), Mira Bela (MBe; state of Minas Gerais), Picos (Pic; state of Piauí), and Pirenópolis (Pir; state of Goiás). Compounds are characterized by GC/EIMS and HPLC/ESIMS.

Compounds		Samples							
		Par	PGr	Bau	Lav	CVe	MBe	Pic	Pir
**1**	4-Vinyl phenol			x	x				
**2**	Dihydrocinnamic acid Methyl ester				x				
**3**	*p*-Vinyl-*O*-prenyl phenol				x				
**4**	2-T-butylnaphto-[2,3-b]-furan,4,9-dione			x	x				
**5**	*p*-Hydroxycinnamic acid methyl ester					x			
**6**	Methyl-*O*-benzoylbenzoate	x							
**7**	Benzene ethoxy	x							
**8**	*n*-Hexadecanoic acid methyl ester							x	x
**9**	Allyl-3-prenylcinnamate			x	x	x			
**10**	2,2-Dimethylchromene-6-propenoic acid methyl ester		x						
**11**	Palmitic acid				x				
**12**	Pinobanksin methyl ether								x
**13**	Oleic acid methyl ester							x	
**14**	4-Hydroxy-3-prenylcinnamic acid methyl ester			x	x	x			
**15**	*n*-Stearic acid methyl ester							x	
**16**	Ferulic acid octadiene ester								x
**17**	Methyl dehydroabietate	x	x						
**18**	*n*-Propyl-*p*-hydroxybenzoate	x							
**19**	12,16-Epoxy-6-hydroxy-(15-16)-abeo-5,8,11,13-abietatetraene-7-one				x				
**20**	Pentyl benzene		x						
**21**	Ferulic acid octaene ester								x
**22**	*n*-Phenyl hexadecane				x				
**23**	Ethyl dehydroabietate	x	x						
**24**	Hydroxy-diprenylcinnamic acid methyl ester (artepillin C)			x	x	x			
**25**	2,2-Dimethyl-8-prenylchromene-6-propenoic acid methyl ester		x	x	x	x			
**26**	Pinobanksin-5-methyl ether acetate			x					
**27**	Benzoic acid, 4(4-hydroxybenzoyl)-methylester		x						
**28**	3-Hydroxy-2,2-dimethyl-8-prenylchromane-6-propenoic acid methyl ester			x		x			
**29**	Benzoic acid, 2-propoxy-(4-hydroxybenzoyl)-methyl ester		x						
**30**	Benzylic alcohol, 2-propoxy-(4-hydroxybenzoyl)-methyl ester		x						
**31**	Benzoic acid, 2(4-hydroxybenzoyl)-methylester		x						
**32**	*n*-Docosanoic acid methyl ester							x	
**33**	*n*-Heptacosane								x
**34**	*n*-Tetracosanoic acid methyl ester							x	
**35**	4-Dihydrocinnamoiloxy-3-prenylcinnamic acid methyl ester			x	x	x			
**36**	*n*-Nonacosane								x
**37**	*n*-Hentriacontane							x	x
**38**	Carbomethoxy benzyl caffeate ester							x	
**39**	Dihydrocaffeoyl-dihydrocinnamoyl-glyceride								x
**40**	*β*-amyrin	x	x		x				
**41**	Propyl carbomethoxy benzyl dihydrocaffeate ester							x	
**42**	Ergosterol								x
**43**	Lupenone								x
**44**	*β*-Amyrinone							x	x
**45**	*α*-Amyrinone							x	x
**46**	**α**-Amyrin								x
**47**	**β**-Amyrin acetate							x	
**48**	**α**-Amyrin acetate					x		x	
**49**	Taraxerone								x
**50**	Oleanene								x
**51**	Pteron-14-en-7-one								x
**52**	Olean-14-en-3,28-dione								x
**53**	Homovanillic acid				x				
**54**	Caffeic acid 4-*O*-glucoside	x	x			x	x	x	x
**55**	Quinic acid		x	x	x	x	x	x	x
**56**	Dihydroquercetin							x	
**57**	Caffeoylquinic acid			x					
**58**	Caffeic acid 4-*O*-arabinoside							x	
**59**	Caffeic acid 4-*O*-xyloside							x	
**60**	Caffeoylquinic acid	x	x		x	x			
**61**	Feruloyl-caffeoylquinic acid			x	x				
**62**	Caffeoylquinic acid				x				
**63**	Methoxy-dihydroxy benzoic acid							x	
**64**	Dimethoxyluteolin-glucoside				x				
**65**	Tricaffeoylquinic acid			x			x		
**66**	Caffeoylquinic acid		x						
**67**	Feruloyl-caffeoylquinic acid			x	x				
**68**	Feruloyl-caffeoylquinic acid			x	x				
**69**	Methylkaempferol-*O*-rutinoside							x	
**70**	Naringenin-*C*-glucoside						x		x
**71**	Apigenin-*O*-rutinoside	x							x
**72**	Feruloylquinic acid					x	x		
**73**	Caffeic acid	x							
**74**	Delphinidin arabinoside						x		x
**75**	Caffeoylquinic acid		x		x	x			
**76**	Catechin arabinoside							x	
**77**	Apigenin-di-*C*-glucosyl rhamnoside						x		
**78**	Apigenin-*C*-rhamnoside								x
**79**	Apigenin-6,8-di-*C*-glucoside (vicenin-2)				x	x	x		x
**80**	Apigenin-*C*-rhamnosyl arabinoside						x		x
**81**	Apigenin-6-*C*-glucosyl-8-*C*-arabinose (isoschaftoside)	x	x		x	x			
**82**	Apigenin-*C*-rhamnoside						x		
**83**	Luteolin-*O*-glucuronide				x				
**84**	Dicaffeoylquinic acid	x	x	x	x	x			
**85**	Caffeic acid-dihydroxy phenyl ethyl ester			x					
**86**	Dicaffeoylquinic acid	x	x						
**87**	Dicaffeoylquinic acid	x	x						
**88**	Apigenin-8-*C*-glucosyl-6-*C*-arabinose (schaftoside)	x	x		x	x			
**89**	Luteolin-6,8-di-*C*-glucoside (lucenin-2)					x			
**90**	Pentosyl orientin					x			
**91**	Vitexin	x				x			
**92**	Quercetin-*O*-glucoside							x	
**93**	Dicaffeoylquinic acid	x	x	x	x	x			
**94**	Ferulic acid-methoxy trihydroxy phenyl ethyl ester			x					
**95**	Hydroxy-prenylcinnamic acid			x					
**96**	3-Prenyl-4-(2-methylpropionyloxy)-cinnamic acid methyl ester			x					
**97**	Luteolin acetyl glucoside	x	x						
**98**	Chrysoeriol-*C*-glucoside					x			
**99**	Dimethoxy naringenin-diglucoside					x			
**100**	Caffeoylferuloylquinic acid	x							
**101**	Apigenin-di-*O*-glucoside					x			
**102**	Quercetin-*O*-arabinoside							x	
**103**	Quercetin-*O*-rhamnoside							x	
**104**	Dicaffeoylquinic acid	x	x		x	x			
**105**	Isorhamnetin-glucoside							x	
**106**	Tricaffeoylquinic acid	x	x		x	x			
**107**	Tricaffeoylquinic acid				x				
**108**	Apigenin-*O*-glucuronide				x				
**109**	Diprenyl chrysin								x
**110**	Ferulic acid dihydroxy phenyl ethyl ester			x					
**111**	Quercetin							x	
**112**	Cinnamoyl hexoside								x
**113**	Caffeoylferuloylquinic acid					x			
**114**	Isorhamnetin							x	x
**115**	Rhamnetin							x	x
**116**	Laricitin								x
**117**	3-Hydroxy-2,2-dimethyl-8-prenylchromane-6-propenoic acid							x	
**118**	Methyl licochalcone B							x	
**119**	Hydroxy-diprenylcinnamic acid (artepillin C)			x					
**120**	Quercetin-dimethyl ether							x	
**121**	Pentamethoxy hydroxy flavonol								x
**122**	Pentamethoxy flavonol								x
**123**	Nobiletin								x
**124**	Chrysin rhamnoside								x
**125**	Prenyl-trimethoxyluteolin								x
**126**	Prenyl-methoxyquercetin								x
**127**	Phloretin								x
**128**	Prenyl-methoxyquercetin								x
**129**	Prenyl-methoxykaempferol					x			x
**130**	Prenyl-dimethoxyquercetin								x
**131**	Prenyl-dimethoxykaempferol								x
**132**	Prenyl-dimethoxykaempferol								x
**133**	Prenyl-trimethoxykaempferol								x
